# Emerging role of liver X receptors in cardiac pathophysiology and heart failure

**DOI:** 10.1007/s00395-015-0520-7

**Published:** 2015-11-26

**Authors:** Megan V. Cannon, Wiek H. van Gilst, Rudolf A. de Boer

**Affiliations:** Department of Cardiology, University of Groningen, University Medical Center Groningen, Hanzeplein 1, 9713 GZ Groningen, The Netherlands

**Keywords:** Cardiac remodeling, Cardiovascular risk factors, Heart failure, Hypertrophy, Liver X receptors, Metabolism

## Abstract

Liver X receptors (LXRs) are master regulators of metabolism and have been studied for their pharmacological potential in vascular and metabolic disease. Besides their established role in metabolic homeostasis and disease, there is mounting evidence to suggest that LXRs may exert direct beneficial effects in the heart. Here, we aim to provide a conceptual framework to explain the broad mode of action of LXRs and how LXR signaling may be an important local and systemic target for the treatment of heart failure. We discuss the potential role of LXRs in systemic conditions associated with heart failure, such as hypertension, diabetes, and renal and vascular disease. Further, we expound on recent data that implicate a direct role for LXR activation in the heart, for its impact on cardiomyocyte damage and loss due to ischemia, and effects on cardiac hypertrophy, fibrosis, and myocardial metabolism. Taken together, the accumulating evidence supports the notion that LXRs may represent a novel therapeutic target for the treatment of heart failure.

## Introduction

The prevalence of heart failure is increasing in our aging society and is associated with significant morbidity and mortality [88]. It is a complex clinical syndrome and diagnosis is based on physical signs and symptoms of dyspnea, fluid retention, and fatigue upon exertion. Heart failure is attributed to a process of pathological cardiac remodeling that is initiated via molecular, cellular, and interstitial changes that structurally and functionally alter the myocardium [54]. Cardiac remodeling is initially a compensatory response to injury such as myocardial infarction or hypertension, but is maladaptive when these factors continue unabated, perpetuating the progression toward cardiac dysfunction and heart failure. The time course and extent of remodeling are influenced by several factors such as the severity of the pathological insult, secondary events including recurrent ischemia or infarction, elevated hemodynamic load, and neurohormonal activation.

**Fig. 1:**
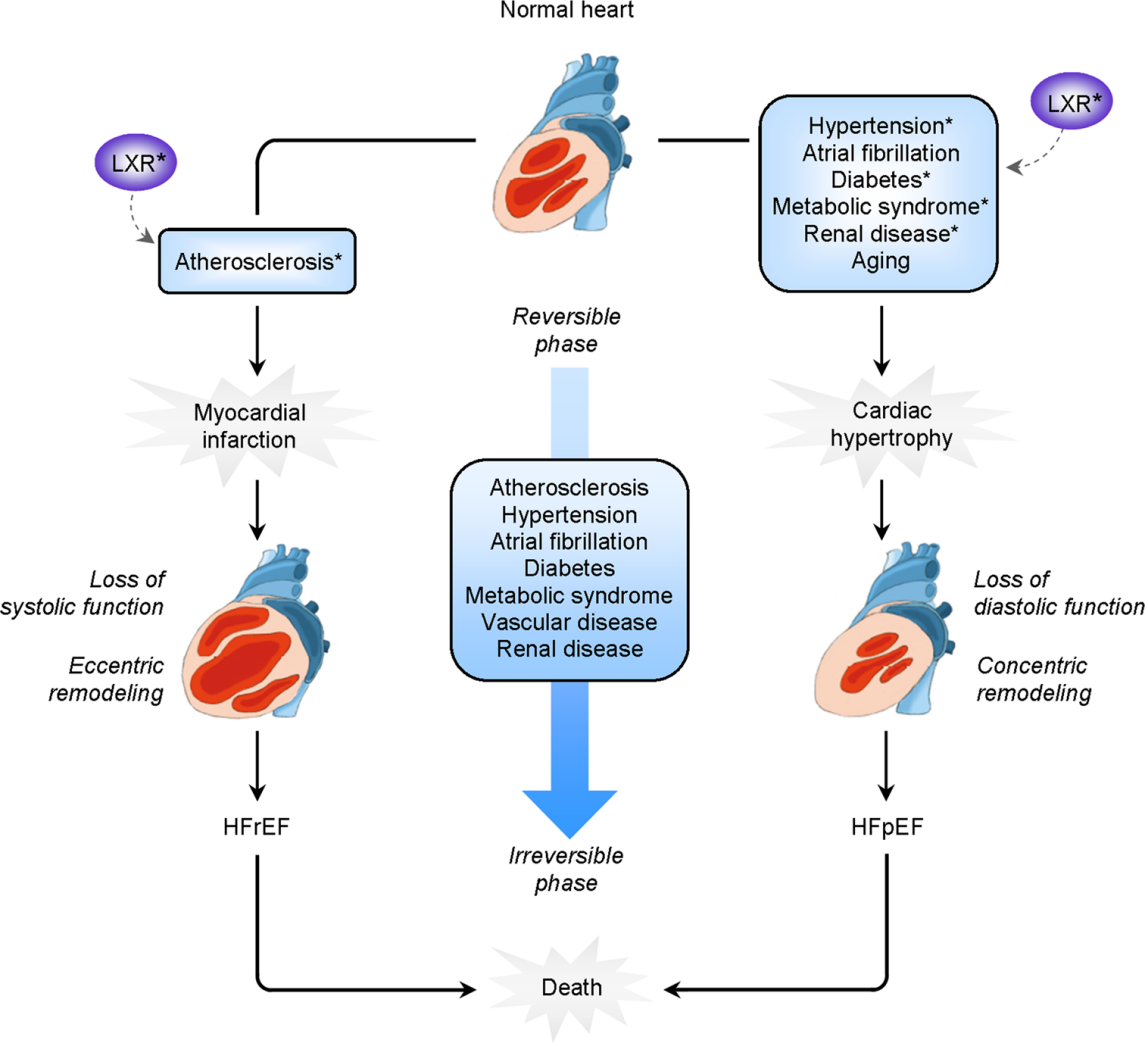
Impact of systemic LXR signaling on co-morbidities associated with heart failure pathogenesis. The progression of atherosclerotic vascular disease increases the risk for myocardial infarction. Myocardial infarction causes eccentric remodeling of the myocardium and loss of systolic function; it is the predominant etiology underlying heart failure with reduced ejection fraction (HFrEF). The presence of co-morbidities such as hypertension, atrial fibrillation, diabetes, metabolic syndrome, renal disease, and aging, all impact the heart, which results in concentric hypertrophic remodeling and loss of diastolic function. This ultimately leads to the pathogenesis of heart failure with preserved ejection fraction (HFpEF). Cardiac remodeling is initially reversible, however, the prevalence of co-morbidities further aggravates the progression of both HFrEF and HFpEF such that adverse remodeling is accelerated and becomes largely irreversible. Consequently, death is inevitable. Liver X receptors (LXRs) are implicated as a protective target in the pathogenesis of heart failure for their beneficial effects in the development of atherosclerosis, insulin resistance and diabetes, nephropathy, and blood pressure modulation*

Heart failure manifests as a state of impaired pump function that results in inadequate cardiac output, and classification is based on left ventricular (LV) ejection fraction which is either reduced (HFrEF) or preserved (HFpEF) (Fig. 1) [46]. The dominant cause of HFrEF is coronary artery disease, and the underlying mechanisms that drive ventricular remodeling are the result of the ischemic injury caused by myocardial infarction. In the infarct zone, a fibrotic scar replaces loss of cardiomyocytes, whereas the remote myocardium undergoes eccentric remodeling that leads to LV dilatation, decreased contractility, and impaired systolic function. In contrast, HFpEF patients demonstrate evidence of diastolic dysfunction, including prolonged LV relaxation time, reduced LV filling, and increased myocardial stiffness [101], and often involves concentric remodeling of the myocardium.

Most pharmacological therapies such as β-blockade and inhibitors of the renin-angiotensin-aldosterone system (RAAS) have proven efficacy in HFrEF by reducing morbidity and mortality [14]. However, this strategy has not been as successful for treating HFpEF [85], therefore diagnosing and treating HFpEF remains a challenge. In essence, the underlying pathophysiology of HFpEF is poorly understood largely owing to the presence of multiple co-morbidities such as hypertension, diabetes, metabolic syndrome, atrial fibrillation, and renal and vascular disease, which impact the pathogenesis of this syndrome and contribute to the complexity of mechanisms governing cardiac remodeling [86]. Lack of a clear understanding of these mechanisms thus poses several challenges in targeting and treating heart failure. Identification of novel pleiotropic targets that are integrative across multi-organ systems may therefore be effective therapeutic strategies in preventing or reversing heart failure.

**Fig. 2:**
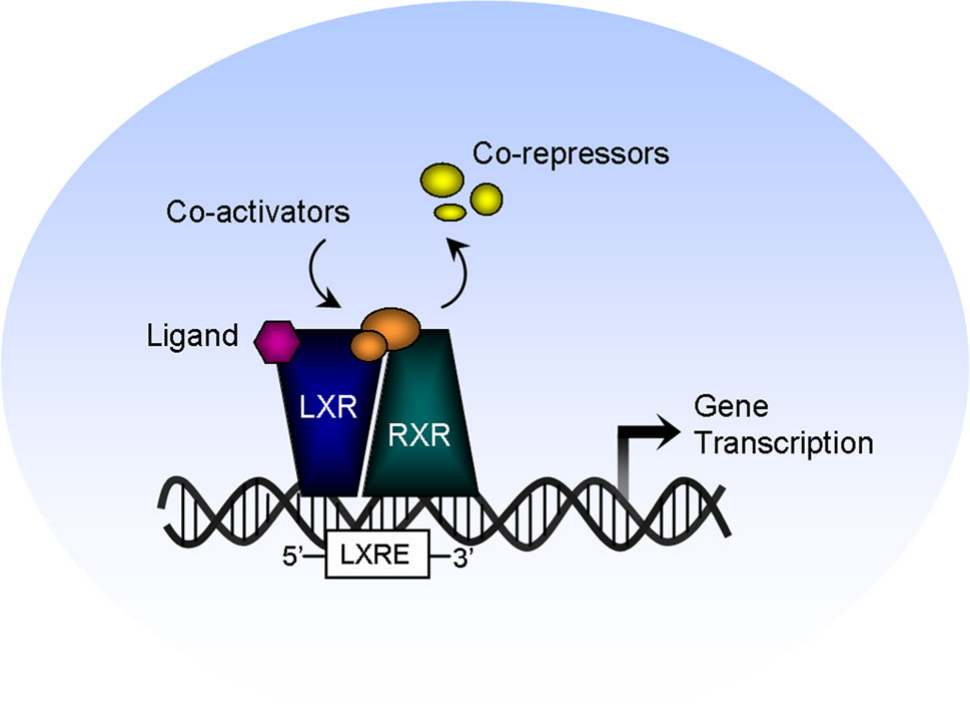
Mechanism of liver X receptor activation. In the nucleus, LXRs form an obligate heterodimer complex with the retinoid X receptor (RXR) that binds to an LXR response element (LXRE) in regulatory regions of target genes. Following ligand binding, the LXR/RXR complex undergoes a conformational change that leads to the release of co-repressors and the recruitment of co-activators, which activates target gene transcription

Liver X receptors (LXR) α and β belong to the nuclear receptor superfamily of ligand-activated transcription factors. LXRs have emerged as important regulators of cholesterol homeostasis, lipid and glucose metabolism, and inflammation, and so have been regarded as promising therapeutic targets for intervention in atherosclerotic and metabolic disease [130]. They were originally discovered in the mid-1990s as “orphan receptors” because their natural ligands were yet unknown [4, 120, 137]. Shortly thereafter, oxysterols as well as cholesterol metabolites were identified as endogenous activators of LXRs, thus LXRs were “deorphanized” [60]. In the nucleus, LXRs form obligate heterodimers with the retinoid X receptor (RXR) and are bound to LXR response elements (LXREs) in regulatory regions of target genes. Binding of either their natural or synthetic ligands, such as T0901317 (T09) and GW3965, induces a conformational change in the LXR/RXR complex that facilitates activation of target gene transcription (Fig. 2). LXRβ is ubiquitously expressed, whereas LXRα features more abundantly in metabolically active systems such as the liver, adipose tissue, and macrophages, as well as in heart, skeletal muscle, kidney, and lung [102]. In the heart, expression levels of both LXRs are 10- to 15-fold higher in the non-myocytic compartment, consisting of fibroblasts and endothelial cells, compared to the myocyte compartment [79].

Here, we review recent evidence regarding the systemic effects of LXRs in relation to co-morbidities that are relevant in heart failure pathogenesis. Although studies of LXRs in the heart are few, they have generated clear evidence that LXR signaling exerts a potential cardioprotective role in cardiac remodeling and myocardial disease. Altogether, we provide a perspective on the potentiality of LXRs as an integrative target for the treatment of heart failure.

## Systemic effects of LXR signaling in the development and progression of heart failure

Combined co-morbidities have a major impact on the pathogenesis of heart failure, which led to the supposition that myocardial dysfunction may not originate exclusively in the heart itself, but extrinsic factors that stem from these co-morbidities may perturb the heart [115]. Here, current knowledge on the role of LXRs in the pathogenesis of several of these co-morbidities, including atherosclerosis and vascular disease, hypertension, diabetes, metabolic syndrome, and chronic kidney disease, is summarized (Table 1). Given the widespread effects of LXRs, we postulate that systemic LXR activation may play an important role in conferring myocardial protection from these disorders that collectively contribute to the pathogenesis of HFrEF and HFpEF.

**Table 1:** Systemic effects of liver X receptor activation in relation to co-morbidities relevant in heart failure pathogenesis

Positive effects of LXR activation			
Atherosclerosis	Hypertension	Diabetes	Chronic kidney disease
↑ Cholesterol efflux [102, 103]	↑ Renin modulation [92, 127]	↑ Glucose tolerance [18, 77]	↓ Albumin:creatinine ratio [72, 99, 126]
↓ Lesion development [64, 128]	↓ AT1R expression [57, 76, 80]	↑ Insulin sensitivity [18]	↓ Lipid accumulation [72, 99]
↓ Inflammation [63]	↑ Vasoreactivity [80]	↓ Endothelial dysfunction [50, 83]	↓ Inflammation [72, 99, 126]
↑ Vascular protection [11] [22]	↓ Blood pressure [75, 80]		↓ Fibrosis [72]

Negative effects of LXR activation			
Lipogenic	Central nervous system		
↑ Liver steatosis [113]	↑ Neurological events [69]		
↑ Hypertriglyceridemia [113]	↑ Psychiatric events [69]		

### LXR and atherosclerosis

Atherosclerosis contributes to multi-organ dysfunction involving the kidney, brain, gut, and skeletal muscle, and is a major cause of HFrEF following myocardial infarction. LXRs have been extensively studied for their putative atheroprotective functions [15]. The initial stages of atherosclerosis involve the formation of foam cells by the uptake of oxidized low-density lipoprotein (LDL) in macrophages in the arterial wall. Mice deficient for both LXRα and LXRβ develop increased foam cell formation, implicating a basal role in cholesterol homeostasis [114]. LXRs limit pathogenic accumulation of cholesterol in macrophages by enhancing the rate of cholesterol efflux [94], which is mediated through upregulation of genes involved in all aspects of the reverse cholesterol transport (RCT) pathway, including Abca1, Abcg1, and ApoE in cellular cholesterol efflux, CETP and PLTP in plasma lipid transport, Abcg5 and Abcg8 for entero-hepatic sterol absorption and excretion, and Cyp7a1 for enhanced bile acid excretion [109]. In murine models, LXR agonist treatment significantly reduced atherosclerosis in both *Ldlr*^−/−^ and a*poE*^−/−^ mice [64], whereas selective loss of macrophage LXR activity through bone marrow transplantations markedly increased lesion development in these models [128]. Interestingly, liver-specific deletion of LXRα in mice leads to decreased RCT, cholesterol catabolism, and excretion while substantially increasing atherosclerosis, altogether underscoring their importance as whole-body cholesterol sensors [143].

In macrophages, the metabolic functions of LXRs are coupled to anti-inflammatory responses, which further contribute to the mechanism underlying their atheroprotective effects. LXR agonist activation inhibits the induction of pro-inflammatory cytokines through a mechanism involving transrepression [63]. In atherogenic *Ldlr*^−/−^ mice, selectively increasing LXRα in macrophages leads to reductions in plasma inflammatory cytokines, IL6 and TNFα, and atherosclerotic lesion development [82]. Moreover, in the absence of cholesterol efflux pathways mediated by *Abca1/g1*-deficient macrophages, LXR agonism nonetheless decreased lesion area, complexity, and inflammatory cell infiltration, including plasma levels of the chemokine, MCP1 [67]. LXRs have also been shown to target the inflammatory process in atherogenesis at a critical step through the inhibition of chemokine-induced CD4-positive lymphocyte migration [133].

More recent evidence implicates additional atheroprotective properties of LXR agonists in the pathogenesis of atherosclerosis that extend beyond their capacity to promote peripheral cholesterol efflux and inhibit inflammation. A novel anti-atherosclerotic mechanism for LXRα through regulation of macrophage iron homeostasis has been identified. By increasing iron export, LXRα reduces iron loading which promotes formation of oxidized lipids, an inducer of cell death [12]. Other functions for LXRs within the vasculature are emerging. Endothelial dysfunction is the underlying cause of all vascular diseases and is a critical initiator of atherosclerosis. LXRs are expressed in endothelial cells [121], and their distribution in murine aortas reveals a greater degree of expression in the atheroprotective thoracic region than in atheroprone areas such as the aortic arch, supporting an anti-atherogenic function [145]. In atherosclerotic vessel walls, studies indicate that LXR activation decreases vascular expression of adhesion molecules such as E-selectin, ICAM-1, and CD44 [131], and improves arterial vasomotor function through enhanced endothelium-dependent vasorelaxation [22].

Also of relevance are the reparative effects of LXRs in vascular injury. The progression of atherosclerosis is accelerated via denuding of the endothelium and intimal injury, which are accompanied by platelet deposition, thrombus formation, and smooth muscle cell proliferation. In rodent models of carotid artery injury, LXR ligands repaired damaged vessel walls by advancing endothelial regeneration through increased proliferation and migration of endothelial progenitor cells and enhancing their secretion of vascular endothelial growth factor (VEGF) [140], as well as inhibiting vascular smooth muscle cell proliferation and neointima formation [11]. Also of note is the identification of a novel role for LXRs in thrombosis and platelet function. Although platelets are anuclear, they reportedly express LXRβ and GW3965 treatment has been shown to inhibit platelet accumulation and thrombi formation [122].

Taken together, evidence of an atheroprotective role for LXRs continues to broaden as studies reveal novel functions in cholesterol efflux, macrophage activity, and vascular protection. Preventing atherosclerotic lesion development is of paramount importance in offsetting coronary syndromes such as myocardial infarction, the major cause of systolic dysfunction leading to HFrEF. Thus, LXRs represent a promising target in the etiology underlying HFrEF and subsequent mortality.

### LXR and hypertension

In HFpEF, hypertension is the most prevalent co-morbidity [89] and precedes heart failure in 60–90 % of all cases [81]. Besides being a risk factor for atherosclerosis and causing vascular injury, hypertension affects the cardiac muscle by invoking pathological hypertrophic growth through increased hemodynamic afterload.

The RAAS is a predominant hormonal signaling pathway in the regulation of blood pressure, fluid balance, and systemic vascular resistance. LXRs have been implicated in blood pressure control through modulation of the RAAS. Initial observations identified LXRα as a regulator of renin transcription [92, 127]. Acute administration of LXR agonists directly increased renin mRNA levels in vivo, whereas LXR-null mice lost their capacity to upregulate renin under β-adrenergic stress [92], suggesting a crosstalk between LXR signaling and the RAAS. In subsequent studies, *chronic* LXR activation inhibited isoproterenol-induced components of the RAAS, including renin, but also angiotensin converting enzyme (ACE) and angiotensin type I receptor (AT1R) expression in kidneys and heart [76]. Furthermore, in vivo investigation of the functional effects of LXRs on RAAS activation revealed that LXR agonism abolished angiotensin (Ang) II-induced increases in blood pressure in rats [80]. Although improved vasoreactivity was not unequivocally linked to the level of RAAS activation, these findings suggest that LXRs decrease peripheral vascular resistance and potentially lower blood pressure. In line with this, the LXR agonist T09 was found to reduce the elevation in blood pressure due to chronic pressure–volume overload in mice, whereas this effect was absent in mice lacking LXRα [75].

The RAAS is not only regulated by mechanisms that stimulate renin release, but is also modulated by natriuretic peptides, ANP and BNP, which are produced by the heart and antagonize the RAAS pathway. Recently, overexpressing cardiac LXRα has been shown to upregulate natriuretic peptide expression [16] (Cannon et al., unpublished data); therefore, LXRα modulation of natriuretic peptides may represent an indirect mechanism for RAAS suppression. Overall, existing evidence suggests that LXRs play a role in antagonizing RAAS activation and may be a viable target in alleviating the hemodynamic burden imposed on the heart.

### LXR and diabetes

Disturbances in energy balance leads to impaired peripheral glucose utilization and the development of insulin resistance and type II diabetes, both of which increase the risk for cardiovascular disease [58]. Diabetes accelerates atherosclerosis, but also directly causes myocardial hypertrophy and diastolic dysfunction in the absence of hypertension or coronary artery disease [43].

LXR agonists have been recognized as a potential pharmacological strategy for the treatment of diabetes and associated metabolic disorders [45]. Multiple studies have established the importance of LXRs in glucose metabolism and in the adaptation to metabolic stress that triggers diabetes. In rodent models of type II diabetes and insulin resistance, LXR agonists have been shown to reduce plasma glucose [18, 84] and improve glucose tolerance and insulin sensitivity [18, 29, 48, 77, 84]. Mechanisms underlying the beneficial effects of LXRs on glucose homeostasis span several organ systems including the liver, adipose tissue, skeletal muscle, and pancreas. In the liver, LXR agonists suppress gluconeogenesis by downregulating Pgc1a, Pepck, and G6Pase genes, and induce glucokinase to promote hepatic glucose utilization [18, 77, 125]. In adipose tissue and skeletal muscle, LXRs directly regulate transcription of the glucose transporter, Glut4, and enhance peripheral glucose uptake both in the absence [33, 77] and presence of diabetes [6, 68]. In pancreatic islet cells, an important homeostatic role for LXRβ has been elucidated as *Lxrβ*^−*/*−^ mice are intolerant to glucose due to impaired glucose-stimulated insulin secretion [44], whereas LXR ligands have been found to promote β-cell insulin secretion [39, 44, 48].

Although LXR agonists represent promising anti-diabetic agents given their insulin-sensitizing effects, the favorable effects on glucose metabolism need to be dissociated from their lipogenic effects for these compounds to be of potential clinical use (Table 1). LXR agonists enhance hepatic and skeletal muscle lipid accumulation and increase circulating triglycerides [30, 68], which worsens the lipogenic pathology in diabetes [26]. Chronic LXR activation may also impair insulin secretion by contributing to lipotoxicity-induced pancreatic β-cell apoptosis [27]. To circumvent these ramifications, alternative approaches are being initiated which include the development of partial LXR ligands or LXRβ-specific agonists since lipogenesis is mediated primarily via LXRα [59]. Interestingly, a recent study reported that administration of the LXR agonist T09 in combination with metformin, an established oral anti-diabetic drug, ameliorated the development of hepatic steatosis induced by LXR agonism in diabetic rats [49], suggesting that combinatorial therapies may also be viable.

Apart from metabolic dysregulation, diabetes is also characterized by low-grade inflammation that stems from macrophage infiltration in adipose tissue and secretion of pro-inflammatory cytokines [129]. By antagonizing NFκB signaling in the nucleus, LXRs have been shown to inhibit the induction of pro-inflammatory genes encoding iNOS, COX-2, IL6, and MCP1 [63]. To date, the impact of the anti-inflammatory functions of LXRs on diabetic pathophysiology is largely unknown. Since chronic systemic inflammation predisposes toward myocardial dysfunction and resultant HFpEF [100], we postulate that LXRs may protect the heart, amongst other susceptible organs, from diabetes- and obesity-induced inflammation.

In summary, LXRs are implicated in the protection against diabetes through modulation of glucose metabolism, β-cell insulin secretion, and inflammatory signaling, including recent developments indicating vasoprotection from hyperglycemia-induced endothelial dysfunction [50, 83]. Overall, LXRs mediate several pathways involved in diabetes, and as such, potentially affect the pathogenesis of HFpEF. These multiple effects are also linked to other co-morbidities contributing to heart failure development: atherosclerosis, the major cause of HFpEF, which is further aggravated by the presence of diabetes, as well as diabetes-induced kidney damage, which results in renal dysfunction, failure, and eventual HFpEF (discussed below).

### LXR, obesity and metabolic syndrome

The metabolic syndrome is comprised of a cluster of metabolic abnormalities that include disturbances in glucose homeostasis, insulin resistance, obesity, dyslipidemia, and elevated blood pressure. These conditions increase the risk of developing cardiovascular disease and diabetes. Maintaining glucose homeostasis and improving insulin sensitivity are important effects of LXRs that potentially influence the development of insulin resistance and diabetes. However, the role of LXRs in modulating the molecular pathogenesis of the metabolic syndrome is less clear.

LXRs are critically involved in cholesterol homeostasis and lipid metabolism. In *Lxrα*^−*/*−^ and *Lxrα/β*^−*/*−^ mice, serum LDL is increased and HDL is decreased, but not in *Lxrβ*^−*/*−^ mice, whereas serum and VLDL and LDL triglycerides are reduced in double-mutants [114]. Further, LXR agonism treatment lowers total and unesterified cholesterol levels in atherogenic *Ldlr*^−*/*−^ mice [64]. In addition to promoting RCT [94], LXRs have also been shown to limit LDL receptor-dependent cholesterol uptake by transcriptionally inducing Idol, an E3 ubiquitin ligase that triggers ubiquitination of the LDL receptor, targeting it for degradation [141].

Apart from enhancing cholesterol catabolism and transport, LXRs are centrally involved in promoting hepatic lipogenesis. LXRs induce lipid synthesis by directly regulating the expression of Srebp1c and downstream target genes, Acc, Fas, and Scd1 [108]. Furthermore, functional LXREs have been identified in the promoter region of ChREBP, a glucose-activated transcription factor that converts excess carbohydrates into lipids [20]. LXRs are also implicated in regulating lipolysis within adipose tissue [124]. In rodents, LXR agonism has been shown to reduce adipocyte size [29], as well as increase serum levels of glycerol and nonesterified free fatty acids, indicative of increased triglyceride hydrolysis [111]. Altogether, dual effects of LXRs on hepatic lipogenesis and adipocyte lipolysis promote hyperlipidemia and insulin resistance, which are undesirable phenomena in the development of the metabolic syndrome.

The effect of LXRs on obesity has also been examined. LXR null mice are resistant to diet-induced obesity and exhibit significant reductions in adipocyte size [65, 74]. In an alternative model of genetic obesity, LXRα/β-deficient *ob/ob* mice remain obese and have increased adipose lipid storage, but display reduced hepatic lipid accumulation and improved insulin sensitivity compared to *ob/ob* mice [7]. Despite being more insulin sensitive, LXRα/β-deficient *ob/ob* mice are, however, glucose intolerant and have impaired pancreatic function. These data suggest that, although LXRs may not protect against obesity, their expression nevertheless influences lipid accumulation, insulin sensitivity, and glucose homeostasis in the setting of obesity [7]. Other studies have shown that LXRs may affect obesity through modulating pathways involved in nutrient status and energy expenditure. LXR agonism downregulated leptin expression in white adipose tissue in mice as well as decreased UCP1 expression, leading to increased energy intake and decreased energy expenditure, respectively [125].

Alternatively, LXRs may protect against obesity through anti-inflammatory functions that ameliorate the development of insulin resistance. LXR agonism has been shown to inhibit TNFα-stimulated release of inflammatory cytokines in fat cells, while re-establishing insulin sensitivity [41]. Thus overall, there is sufficient evidence to suggest that LXRs modulate key components of the metabolic syndrome.

### LXR and chronic kidney disease

Nephropathy is a microvascular complication of diabetes mellitus and uncontrolled hypertension, leading to chronic kidney disease [112]. These, and other causes of chronic kidney disease, are major contributors to cardiac damage and are associated with an increased risk for cardiovascular disease [13, 117].

LXRs have been implicated as a renoprotective target, preserving intrinsic renal structure and function both basally and in diabetic nephropathy. A homeostatic role for LXRs in kidney function has been postulated. *Lxrβ*^−*/*−^ mice exhibit polyuria and polydipsia, features of diabetes insipidus [42], and mice deficient for both LXRs display a renal phenotype analogous to diabetic nephropathy with elevations in albumin:creatinine ratio and glomerular lipid accumulation [99]. When challenged with diabetes, these mice demonstrated accelerated mesangial matrix expansion, increased glomerular lipid, and upregulation of inflammatory and oxidative stress markers [99].

In the kidney, expression levels of both LXRs are significantly decreased in animal models of type I diabetes [105] and in patients with diabetic nephropathy [87]. Studies conducted in several diabetic rodent models demonstrated that LXR activation with T09 and GW3965, as well as a new generation agonist, N,N-dimethyl-3β-hydroxycholenamide (DMHCA), prevented renal damage and dysfunction by reducing urinary albumin excretion and inhibiting macrophage infiltration, inflammation, and lipid accumulation [72, 99, 126]. Besides local renal effects, macrophage-derived LXR signaling is also pertinent in renal pathophysiology as transgenic LXRα overexpression in macrophages protected from hyperlipidemic-hyperglycemic nephropathy [72]. These findings suggest that LXRs play an important role in hyperglycemic-induced kidney disease. Whether LXRs affect hypertension-associated renal impairment remains to be established.

## Local cardiac effects for LXRs

**Fig. 3:**
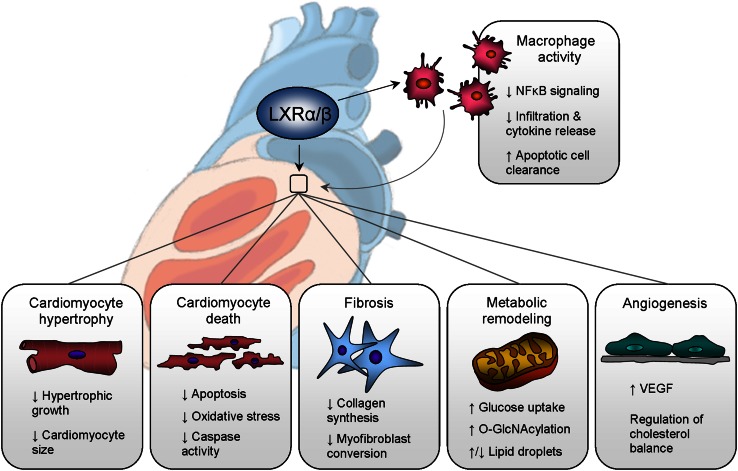
Cardiac effects of liver X receptor signaling. In the ischemic or hypertrophic myocardium, LXRs decrease cardiomyocyte hypertrophy and loss (death), as well as attenuate fibrotic remodeling. LXRs also modulate myocardial metabolism, and have regulatory functions in angiogenesis and neovascularization. LXRs are also expressed in macrophages, which can affect the myocardium through injury or disease. NFκB, nuclear factor kappa B; VEGF, vascular endothelial growth factor

Relatively few studies have evaluated LXR signaling in the heart itself. Both LXRα and LXRβ are expressed in the heart where in comparison to the cardiomyocyte fraction, their expression levels are 10- to 15-fold higher in the non-myocytic fraction consisting of fibroblasts and endothelial cells [79]. In the heart, LXRs are induced and activated by myocardial infarction [51, 79], chronic pressure overload [17, 139], myocarditis [98], and diabetes [25, 52], indicating that LXRs are regulated in cardiac (patho)physiology. Here, we review current evidence regarding local cardiac effects for LXRs and the potential role of LXR signaling in the fundamental cellular processes governing cardiac remodeling, including cardiomyocyte hypertrophy and death, fibrosis, and metabolic and vascular remodeling (Fig. 3).

### Cardiomyocyte hypertrophy

Cardiomyocytes are non-proliferative, therefore they respond to pathophysiological stimuli through hypertrophic growth in order to reduce ventricular wall stress and augment contractile function. Increases in mechanical load as well as various neurohumoral signals such as catecholamines, growth factors, cytokines, and vasoactive peptides, activate a cascade of signal transduction pathways within the myocyte that orchestrates transcriptional reprogramming and posttranslational modification of protein synthesis and growth, as well as reactivation of fetal contractile and metabolic gene expression (fetal gene program). Initially, these adaptations are compensatory and considered protective, but with unremitted stress, become maladaptive and yield to pathological concentric or eccentric growth.

Several lines of evidence suggest an important role for LXRα in regulating hypertrophic cardiac remodeling. First, from in vitro experiments, LXR agonists have been shown to decrease cellular hypertrophy induced by diverse hypertrophic stimuli such as Ang II and lipopolysaccharides [139], endothelin-1 [75], and phenylephrine [17], whereas knockdown of LXRα in cardiomyocytes led to increased cellular growth [17]. Second, LXRα protein abundance is markedly upregulated in the pressure overloaded myocardium [17, 139]. Third, murine hearts deficient for LXRα demonstrate an exacerbated hypertrophic response to chronic pressure overload induced via transverse aortic constriction (TAC) [139], whereas the LXR agonists T09 and AZ876 attenuate hypertrophy in wild-type mice [17, 75], but not in LXRα-null mice [75]. Finally, the effect of LXRα on cardiac hypertrophy is heart-specific and independent of confounding systemic effects attributed to either T09 activation, which are lipogenic [102], anti-inflammatory [142], and blood pressure-lowering [75] effects, or residual effects from whole-body LXRα deletion [73]. Cardiac-specific LXRα overexpression in transgenic mice protected the heart from TAC-induced pressure overload and Ang II stimulation by attenuating LV hypertrophy and preventing cardiac dysfunction, in both the early and later phases of LV remodeling [16]. Taken together, these findings suggest a cell-specific role for LXRs in cardiomyocyte hypertrophy and are supported by pharmacological and genetic studies.

The molecular basis for the antagonistic actions of LXRα on pathological hypertrophic growth has been elucidated. In cardiomyocytes, suppressed pro-inflammatory NFκB signaling was found to be operative in T09-mediated decreases in cellular growth [139]. There are, however, various stages in the development of pathological cardiac hypertrophy, and the induction of an inflammatory response is only an early manifestation of this progression. Further evidence indicates that, in the intermediate phase of hypertrophy prior to decompensation and heart failure, the cardioprotective effects of LXRα may involve transcriptional regulation of myocardial glucose metabolism in the adaptation to hypertrophic stress. Mice with cardiac LXRα overexpression display an endogenous protective phenotype evidenced by enhanced myocardial glucose uptake that is linked to induction of potent anti-hypertrophic effectors, ANP and BNP. Specifically, increases in glucose flux activate the hexosamine biosynthetic pathway, which leads to downstream posttranslational O-GlcNAcylation of transcription factors of natriuretic peptides, GATA4 and Mef2c, potentiating their activities [16].

### Cardiomyocyte death

Loss of cardiomyocytes is crucial in the pathogenesis of myocardial infarction, ischemia/reperfusion (I/R), and heart failure. Since cardiomyocytes are terminally differentiated and generally incapable of replicating in the adult heart, their survival is critical for maintaining myocardial viability. Cell death arises when blood supply to the myocardium is disrupted by coronary occlusion. Ensuing hypoxic and ischemic stimuli increase ROS production which triggers necrosis and apoptosis.

Recently, a role for LXR in apoptosis has been evaluated in the infarcted heart. LXR agonist treatment decreased infarct size and improved LV contractile function in murine hearts subjected to global I/R injury, and prevented hypoxia-reoxygenation-induced apoptosis by attenuating caspase 3 [79]. Furthermore, LXRα, but not LXRβ, was identified as the predominant isoform in the protection against acute and chronic I/R injury, which was associated with significant reductions in post-ischemic myocardial apoptosis. These infarct-sparing effects occurred via inhibition of endoplasmic reticulum stress- and mitochondria-mediated apoptotic pathways through targeting of caspases 12 and 9, respectively [51]. In an alternative therapeutic approach for cardiac repair following myocardial infarction, the combined therapy of the LXR agonist T09 and adipose-derived mesenchymal stem cells (AD-MSCs) transplanted into infarcted hearts inhibited host cardiomyocyte apoptosis and improved cardiac function, while T09 further improved the survival of AD-MSCs under hypoxic conditions [135]. Altogether, these findings are consistent with other studies demonstrating protective effects for LXRs in ischemic models of intestinal and brain injury, although the mechanisms underpinning LXR efficacy in these studies are linked to suppressed pro-inflammatory NFκB signaling [23, 31, 118].

LXRs have also been shown to regulate cell survival through inhibition of ROS production and oxidative stress [47, 121], as well as prevent apoptosis induced by hyperglycemia [24] and diabetes [52]. Interestingly, the anti-apoptotic factor AIM, also known as apoptosis inhibitor 6 or Spα, is a direct target gene for regulation by LXRα [62], which serves to protect macrophages from the apoptotic effects of oxidized lipids. Moreover, macrophages are essential for wound healing following myocardial infarction as they are the primary initiators of phagocytosis of the apoptotic cell. The inability to clear apoptotic cells is pro-inflammatory, and LXR signaling has been demonstrated to play an important role in apoptotic cell clearance and enhanced macrophage phagocytosis [1, 107]. In summary, LXRs may modulate critical facets of cellular death pathways in the ischemic and infarcted myocardium.

### Fibrosis

Cardiac fibrosis is central in the pathogenesis of heart failure and is a major constituent in LV remodeling resulting from myocardial infarction and chronic pressure overload. With myocardial infarction, cardiomyocyte loss is resolved by cardiac fibroblasts, which initiate wound healing through replacement fibrosis to form a scar resilient in preventing ventricular rupture. Fibrosis that develops from pressure overload and in remote regions after myocardial infarction is termed reactive fibrosis. This form of fibrosis interferes not only with transduction of electrical impulses and contractile force in myocytes, but impedes diastolic relaxation through increased myocardial stiffness. In response to pathological stimuli, fibroblasts proliferate and differentiate into myofibroblasts, which contract and secrete collagens. Increased collagen deposition as well as changes in the balance of proteins regulating extracellular matrix turnover disrupt normal matricellular architecture. To date, there are no therapeutic strategies that specifically target fibrogenesis in the heart.

LXR agonists exhibit anti-proliferative properties as demonstrated in pancreatic β-cells [90], smooth muscle cells [11, 37], lymphocytes [9], and cancer cells [28]. Both LXRs are expressed in cardiac fibroblasts, and thus in the heart, they may potentially reduce expansion of the fibroblast population in LV remodeling. LXR activation with the novel agonist AZ876 resulted in both suppressed myofibroblast conversion and the prevention of Ang II- and transforming growth factor β (TGFβ)-induced collagen synthesis [17]. Further, in murine models of chronic pressure overload and diabetes, LXR agonism has been shown to oppose increases in myocardial fibrosis and pro-fibrotic gene expression in conjunction with improved diastolic function [17, 52]. Cardiac-specific LXRα overexpression also exerts anti-fibrotic effects and lowers fibrotic gene expression [16]. These observations are in accordance with other studies demonstrating anti-fibrotic effects of LXRs in liver injury [8], diabetic nephropathy [72, 126], and experimental skin fibrosis [10]. The latter two findings are associated with the interference of LXR ligands in macrophage infiltration and release of cytokines. Given that multiple co-morbidities contribute to a sustained pro-inflammatory state particularly evident in HFpEF [136], these effects on macrophage activity and fibrosis are of clinical relevance. Also of note, LXRs have been reported to downregulate matrix metallopeptidase MMP9 via repressed NFκB signaling [19], suggesting a role for macrophagic LXRs in regulation of extracellular matrix turnover.

LXRs may counteract pro-fibrotic signaling in the heart by affecting key intracellular pathways. For example, Ang II signaling promotes fibroblast proliferation and extracellular matrix formation through AT1 receptor-dependent activation. Several studies have demonstrated downregulation of AT1R by LXR agonism [57, 76, 80] through dephosphorylation of the transcription factor, Sp1 [57]. TGFβ is another central mediator of multiple inflammatory and fibrotic cellular responses in cardiac remodeling [38]. A crosstalk between LXRs and TGFβ signaling via interaction with the co-activator RAP250 and Smad2/3 has been determined [3]. Furthermore, LXR agonism has been shown to attenuate TGFβ and downstream Smad2/3 expression in hypertrophied murine hearts [17], which is consistent with their activation in other diseased states such as diabetes-induced renal fibrosis [126] and chronic asthma-induced airway remodeling [116]. These findings altogether implicate a role for LXRs in fibrogenesis, raising the possibility that they may serve as a protective target in the prevention of fibrosis in cardiac remodeling.

### Metabolic remodeling

The heart is a highly metabolic organ requiring continuous replenishment of its cellular ATP stores to support sarcomeric contraction and relaxation as well as functioning of membrane transport systems. The demand for energy is supplied primarily through oxidation of fatty acids, but also glucose and to a lesser extent lactate, ketones, and amino acids. However, pathological states trigger alterations in myocardial substrate utilization in order to maximize energy efficiency through reciprocal downregulation of fatty acid oxidation and enhanced glucose reliance. As heart failure progresses, myocardial metabolism in maladaptive as oxygen and substrate supply are decreased and energy transfer is impaired, resulting in mitochondrial dysfunction and inefficient energy utilization. In essence, the failing heart becomes “an engine out of fuel” [95] as energy production is inadequate to support cardiac output.

Promoting the shift towards increased glucose reliance has been postulated to improve myocardial efficiency in heart failure, preventing energy depletion [5, 61]. LXRα has been implicated in the transcriptional regulation of cardiac glucose metabolism and orchestration of an adaptive metabolic response to hypertrophic stress. Constitutive LXRα overexpression in isolated cardiomyocytes and murine hearts induced Glut1 and Glut4 expression and increased the capacity for glucose uptake and utilization. Furthermore, mice deficient for LXRα display impaired myocardial glucose uptake in response to hypertrophic perturbation, whereas glucose levels are substantially enhanced with cardiac LXRα overexpression in the protection against cardiac dysfunction [16]. Interestingly, LXRα has also been shown to improve long-term cardiac performance following chronic I/R injury that is evidenced in part by preserved glucose uptake [51], an indicator of myocardial viability in this setting. Whether preservation of glucose uptake was a direct effect of LXRα on glucose metabolism, or secondary to reduced infarct size was not established herein. In contrast, lipid droplet accumulation has been observed following treatment with the LXR agonist GW3965, which associated with improved tolerance to acute myocardial ischemia [79]. At the onset of I/R there is increased availability and usage of fatty acids [66]. However, the role of lipids in the heart remains controversial as it may lead to lipotoxicity and dysfunction [97]. Glucose pathways were not assessed in this study and therefore cannot be precluded as a protective mechanism against acute myocardial infarction.

Targeting LXRα to promote cellular glucose uptake may indeed be advantageous under conditions where glucose uptake is compromised. For example, insulin resistance is highly prevalent in the diabetic and heart failure population and can impair the shift to glucose reliance [13, 93, 138]. LXRα may therefore serve as an important tool in sensitizing the heart to glucose under these conditions. Recently, LXR agonism has been shown to protect against diabetic cardiomyopathy in *db/db* mice, although the effects on cardiac metabolism were not investigated in this study [52]. An alternative investigation in mice overexpressing cardiac LXRα revealed that the elevated capacity for myocardial glucose uptake is sustained despite obesity-induced systemic insulin resistance and hypertriglyceridemia (Cannon et al., unpublished data), stimuli that impede the capacity for glucose uptake. Overall, LXRs represent a potential metabolic modulator for optimization of myocardial substrate utilization in cardiac pathophysiology.

### Angiogenesis

Endothelial dysfunction involves a range of endothelial cell functions that become dysregulated, including impaired angiogenic responses that are crucial in salvaging the infarcted and hypertrophic myocardium [35]. In HFrEF, new microvascular networks are necessary for improving regional perfusion after ischemic injury. In HFpEF, muscle-to-capillary ratio is reduced in pathologically hypertrophied hearts, which causes myocardial hypoxia and contractile dysfunction through compromised oxygen and nutrient delivery that is necessary for growth. The aim of cardiovascular therapies is to stimulate angiogenesis within the myocardium to prevent or reverse heart failure.

The angiogenic potential of LXRs has been established in the ischemic rat brain [118] and following stroke [21]. LXRs promote angiogenesis by directly regulating VEGF [134]. Murine and human Vegfa genes harbor a functional LXRE in the promoter region, and regulation occurs independent of the hypoxia response element for HIF-1 [134]. However, several studies provide evidence for an anti-angiogenic (yet protective) role for LXRs in settings of uncontrolled angiogenesis such as in tumor growth [96] and cancer [36, 104]. Whether LXRs stimulate angiogenesis in either the ischemic or hypertrophic myocardium remains to be determined.

LXRs may also regulate angiogenesis through modulation of cholesterol. Cholesterol promotes lipid raft formation in the plasma membrane from where cell surface receptors initiate signaling events that lead to angiogenesis [40]. Since excess cholesterol or its removal determines the balance between either promoting or inhibiting angiogenesis, this balance may arguably be regulated by LXRs given their antagonistic actions on increasing HDL cholesterol [113] and stimulating cholesterol efflux via the RCT [94]. These data implicate a role for LXR in angiogenesis and suggest that LXRs may prevent tissue hypoxia, either through directly targeting VEGF or through modulation of cholesterol.

## Conclusions

Heart failure is a complex clinical syndrome, and co-morbidities such as hypertension, diabetes, and kidney and vascular disease, including atherosclerosis, are increasingly recognized for their provocation of this disease. Left untreated, these co-morbidities accelerate the progression of adverse myocardial remodeling which results in impaired cardiac function, eventually leading to heart failure and death. The optimal window for therapeutic modulation likely occurs in the early phase when cardiac remodeling is largely reversible. Since systemic LXR activation may exert many roles in the pathogenesis of the aforementioned co-morbidities, targeting of LXRs during this phase may serve as a useful addendum in preventing remodeling progression. In addition, accumulating evidence implicates intracardiac LXR signaling in the protection against pathological processes involving myocyte hypertrophy and loss, fibrosis, and metabolism. However, translation of basic findings to clinical settings requires additional studies to further elucidate the role of LXRs in the heart. In the presence of multiple co-morbidities, the collective impact of systemic LXR activation on preserving myocardial integrity and function warrants further address.

Thus far, only few data exist on the role of LXR signaling in humans. In a human genetic study, analysis of the LXR gene sequence in patients afflicted with coronary artery disease revealed mutations in the ligand-binding domain of LXRα that altered its conformation, rendering LXRα inept in binding its ligands [34]. This suggests that humans having a mutated LXRα gene may develop a predisposition towards coronary artery disease. In further support of this, an LXRα gene polymorphism was found to be related with increased susceptibility of coronary artery disease in Chinese Han population [144]. Genetic variation in the LXRα-encoding gene *NR1H3* has been associated with increased risk of ischemic vascular disease [123], as well as LV hypertrophy (Van der Harst et al., unpublished data), obesity [32, 110], longevity [91], and the metabolic syndrome [78], although another study was not able to validate the latter [110]. Polymorphisms in the *NR1H2* gene encoding LXRβ mainly associated with obesity and type 2 diabetes [32, 70, 119]. Interestingly, the LXR signaling pathway was recently found to be highly enriched among pleiotropic genes that emerged from cardiovascular disease risk factor clustering, and for coronary artery disease [106], supporting the notion that the LXR pathway may affect many cardiometabolic traits.

## Future perspectives

Testing of high affinity agonists specific for LXRs are needed to further evaluate the potential of LXR activation in human disease, and provide more appreciable insight into their therapeutic prospective [55]. To date, only one clinical study has been published for the LXR agonist, LXR-623, which activated LXRs without causing hepatic lipogenesis. However, this clinical trial was prematurely halted due to adverse neurological side effects in subjects tested [69]. Nevertheless, several other LXR agonists are in development and natural modulators of LXR activity are also being considered for their potential as nutraceuticals for therapy [56]. Recently, a novel partial, LXRβ-selective agonist, BMS-779788, has been shown to display an improved plasma lipid profile compared with a dual agonist in a cynomolgus monkey model, while inducing genes that enhance the RCT [71]. Indeed, much of the current interest in developing new generation compounds is channeled towards designing LXRβ-specific ligands that target induction of the RCT pathway and circumvent the hepatic lipogenic signaling pathways mediated by LXRα. However, in the heart, there is mounting evidence to suggest that LXRα is the predominant isoform in the protection against distinct myocardial pathologies related to ischemia [51], pathological hypertrophy [16, 17, 139], and diabetic cardiomyopathy [25]. Ideally, a tissue-selective LXR agonist with high affinities for cardiac LXRα may be more suitable for treating myocardial disease. A strategy to exclusively target LXRα pathways in a given cell, or tissue, such as in the heart, is to modulate the LXR co-regulatory transcriptional complex, which impacts its DNA-binding and transcriptional activity [2, 53, 132]. Hence, a greater understanding of the co-factor profile recruited to regulate LXRα in the normal and diseased heart may aid in this development.

In perspective, bridging the gap between preclinical studies and a clinically viable therapeutic strategy most likely lies in the development of highly specific and selective LXR ligands that minimize lipogenic and neurological side effects, and are well tolerated in clinical trials. Given the global salutary effects of LXR activation in cardiovascular disease and its precursors, atherosclerosis, hypertension, diabetes, and inflammation, we postulate that effective and successful targeting of LXRs holds promise for future therapies.
